# Intraspecific trait plasticity to N and P of the wetland invader, *Alternanthera philoxeroides* under flooded conditions

**DOI:** 10.1002/ece3.9966

**Published:** 2023-03-31

**Authors:** Nathan E. Harms, Ian A. Knight, A. Blake DeRossette, Dean A. Williams

**Affiliations:** ^1^ Aquatic Ecology and Invasive Species Branch US Army Engineer Research and Development Center 201 E. Jones St. Lewisville Texas 75057 USA; ^2^ Aquatic Ecology and Invasive Species Branch US Army Engineer Research and Development Center 3909 Halls Ferry Rd. Vicksburg Mississippi 39180 USA; ^3^ Department of Biology Texas Christian University Fort Worth Texas USA

**Keywords:** adaptive evolution, biogeography, biological control, invasive species, latitudinal pattern, nutrient enrichment, phenotypic plasticity, wetland invader

## Abstract

Interactions between invaders and resource availability may explain variation in their success or management efficacy. For widespread invaders, regional variation in plant response to nutrients can reflect phenotypic plasticity of the invader, genetic structure of invading populations, or a combination of the two. The wetland weed *Alternanthera philoxeroides* (alligatorweed) is established throughout the southeastern United States and California and has high genetic diversity despite primarily spreading clonally. Despite its history in the United States, the role of genetic variation for invasion and management success is only now being uncovered. To better understand how nutrients and genotype may influence *A. philoxeroides* invasion, we measured the response of plants from 26 *A. philoxeroides* populations (three cp haplotypes) to combinations of nitrogen (4 or 200 mg/L N) and phosphorus (0.4 or 40 mg/L P). We measured productivity (biomass accumulation and allocation), plant architecture (stem diameter and thickness, branching intensity), and foliar traits (toughness, dry matter content, percent N, and percent P). A short‐term developmental assay was also conducted by feeding a subset of plants from the nutrient experiment to the biological control agent *Agasicles hygrophila*, to determine whether increased availability of N or P to its host influenced agent performance, as has been previously suggested. *Alternanthera philoxeroides* haplotype Ap1 was more plastic than other haplotypes in response to nutrient amendments, producing more than double the biomass from low to high N and 50%–68% higher shoot: root ratio than other haplotypes in the high N treatment. *Alternanthera philoxeroides* haplotypes differed in seven of 10 variables in response to increased N. We found no differences in short‐term *A. hygrophila* development between haplotypes but mass was 23% greater in high than low N treatments. This study is the first to explore the interplay between nutrient availability, genetic variation, and phenotypic plasticity in invasive characteristics of the global invader, *A. philoxeroides*.

## Graphical abstract

Interhaplotype trait differences may explain spatial variation in invasion and management success of invasive plants. We compared three common haplotypes of the wetland weed, *Alternanthera philoxeroides*, and found that Ap1 is more responsive to elevated nitrogen, but not phosphorous, than Ap3 and Ap6. Ap1 produced more biomass overall and allocated a larger proportion to aboveground structures, but we found no differences in herbivory related to plant haplotype. These differences may explain the larger geographic distribution of Ap1 than other haplotypes in the United States.

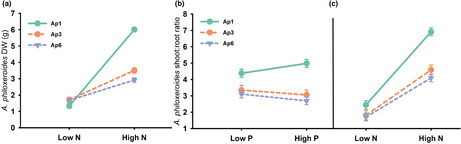

## 1 INTRODUCTION

Invasive species are recognized as major economic and ecological hazards, with the frequency and magnitude of their negative impacts expected to increase in the future (Diagne et al., 2021). Determining the influence of local site conditions on invader establishment, spread, and abundance is of paramount importance for prioritizing areas for management and predicting impacts associated with invasions (e.g., Godoy et al., 2011; Kolb & Alpert, 2003; Woo & Zedler, 2002). Studies that examined invader response to competition or nutrient enrichment have advanced our understanding of traits associated with successful invaders and provided research directions to develop effective conservation plans, with the goal of reducing the likelihood of impacts and increasing species diversity overall (Buckley, 2008; Larson et al., 2011).

Within aquatic and wetland ecosystems, increased availability of nitrogen and phosphorus (i.e., eutrophication) has emerged as a key variable in a number of successful invasions and has attracted research focus to mitigate its prevalence globally (Carson et al., 2018; Gérard et al., 2014; Scherer‐Lorenzen et al., 2008). The role of nutrient enrichment in plant invasions has been examined for a number of systems (e.g., Fan et al., 2013; Yu et al., 2018) and in addition to increasing the likelihood of negative impacts caused by the invader, elevated nutrient levels can impact when and how management is applied (Elgersma et al., 2017; Room et al., 1989). For example, successful biological control of weeds with insects can be highly dependent on plant nutrition, with significant effects of nitrogen on the timing of agent establishment (Room & Thomas, 1985), population buildup (Harms & Cronin, 2019; Harms, Cronin, et al., 2020), and dispersal (Wilson et al., 2007). The relationship between eutrophication and herbicide efficacy is less clear, but there are numerous examples of increased herbicide efficacy when plants are grown with supplemental fertilization before application (Aulakh, 2020; Cathcart et al., 2004).

Invader response to fertilization or management may depend on genetic structure of invasions, resulting from sexual recombination or the introduction and establishment of populations from multiple native range source locations (Ward et al., 2008). The ways in which genetic variation and eutrophication interact during biological invasions is largely unstudied but has begun to receive interest (Harms, Cronin, & Gaskin, 2021; Holdredge et al., 2010; Kettenring et al., 2011; Liao et al., 2016), especially with ever‐increasing nutrient inputs to natural systems (Hale et al., 2015). For example, the aquatic invasive plant flowering rush (*Butomus umbellatus* L.) has multiple genotypes and at least two cytotypes in the United States (Kliber et al., 2005), and previous work demonstrated that diploid and triploid populations differed significantly in their growth, resource allocation, and chemical response to N or P (Harms, Cronin, & Gaskin, 2021). In the southeastern United States, the aquatic plant *Alternanthera philoxeroides* (Mart.) Griseb. has been introduced at least twice (Kay & Haller, 1982), with introduced biotypes displaying differential susceptibility to herbicides (Kay, 1992) and to biological control agents (Pan et al., 2012). Despite considerable interest in managing the spread and establishment of *A. philoxeroides*, the possible interaction between genetic variation within invading populations and nutrient response and allocation of plants is unknown.

To better understand how genetic variation and nutrient availability interact to promote invasions and their management, we conducted a common garden experiment with the aquatic invader, *A. philoxeroides*. We grew plants under combinations of low or high nitrogen and phosphorus, then measured (1) key growth, structural, and elemental chemistry traits, and (2) short‐term performance of biocontrol agents fed leaves of plants grown under different nutrient combinations. Based on previous studies, we expected the broad‐stemmed morphotype (Ap1 sensu Williams et al., 2020) to display the greatest growth response to increased nutrient levels. We additionally expected elevated nutrients to translate into higher tissue nutrients and faster development of the alligatorweed flea beetle. This is the first study to link genetic variation in a widespread clonal invader, invader performance and phenotype, and management with biological control.

## 2 MATERIALS AND METHODS

### 2.1 Study system

*Alternanthera philoxeroides* is an aquatic macrophyte native to South America, which has been introduced in over 32 countries around the world (Tanveer et al., 2018). *Alternanthera philoxeroides* is a common and widespread invader in the Southeastern United States but there are disjunct populations in California (Walden et al., 2019). There are at least six haplotypes of alligatorweed in the United States, likely from multiple introductions (Williams et al., 2020). The biological control program for alligatorweed consists of three insect agents (alligatorweed thrips, *Amynothrips andersoni* O'Neill; alligatorweed moth, *Macrorrhinia endonephele* [Hampson] [= *Arcola malloi* Pastrana]; alligatorweed flea beetle, *Agasicles hygrophila* [Selman and Vogt]), which were introduced in the late‐1960s and early‐1970s (Buckingham, 1996). The most common agent in the United States is *A. hygrophila* but low winter temperatures limit its distribution to warm southern portions of the range of *A. philoxeroides* (Harms & Cronin, 2020).

The introduction history of *A. philoxeroides* into the United States is unclear. *Alternanthera philoxeroides* is thought to have been first introduced into the country via ship ballast water release and was recorded in Alabama and Louisiana prior to 1900 (Zeiger, 1967). However, there are no subsequent records of introductions since then, and any introductions that may have occurred would have been cryptic. As early as 1982, it was clear that multiple genotypes of *A. philoxeroides* were present, with differences in traits between them (Kay & Haller, 1982). It has been further acknowledged that multiple introductions likely occurred but, until recently, the degree of genetic diversity and its role in invasive plant management in the United States was unknown (Geng et al., 2016; Williams et al., 2020).

### 2.2 Plant culture

Alligatorweed clones were collected from 26 locations across the entire US distribution (Table 1) and cultured year‐round in the BioManagement greenhouse at the US Army Engineer Research and Development Center (ERDC), Vicksburg, Mississippi, the United States. Populations were originally collected during 2017–2018 and propagated repeatedly under identical conditions to reduce the role of collection site conditions on plant phenotype (Roach & Wulff, 1987; Wolf & Wade, 2009). Plant culturing consisted of growing plants hydroponically in 20‐L plastic buckets with 16‐L half‐strength Hoagland's nutrient solution (Hoagland & Arnon, 1950). During the growing season and leading up to experiments, nutrients were exchanged biweekly. At other times, nutrients were exchanged monthly. Plants were periodically pruned and thinned to maintain low culture biomass when not needed, and pests (e.g., whiteflies, prob. *Trialeurodes* sp.; aphids, *Myzus persicae*) were treated with insecticidal soap as they were observed. Pest control continued throughout the year but ceased within 4 weeks of experiments, and fresh pest‐free cuttings were used to start experiments.

**TABLE 1:** Source populations, chloroplast haplotype, and original collection coordinates of plants used in this study.

Site name	Cp haplotype	Latitude	Longitude
Lake Monroe, FL	Ap1	28.834508	−81.3224306
Navidad River, TX	Ap1	29.035598	−96.563091
Newnan's Lake, FL	Ap1	29.617992	−82.2534528
Choctaw Boat Ramp, LA	Ap1	29.84985	−90.67883
Lake Waco, TX	Ap1	31.609716	−97.304721
Cooter's Pond, AL	Ap1	32.43119	−86.39978
Longbranch, MS	Ap1	33.7689	−90.1442
TennTom Waterway, MS	Ap1	33.661111	−88.487222
Aberdeen Lake, MS	Ap1	33.825114	−90.5
Lake Merrisach, AR	Ap1	34.032873	−91.26608
333 Cove, AR	Ap1	35.3204	−93.214
Suwanee, FL	Ap3	30.30096	−82.93193
Valley Park, MS	Ap3	32.6347	−90.8632
McGehee, AR	Ap3	33.63486	−91.38953
Lake Wallace, SC	Ap3	34.63038	−79.68023
Lake Marion, SC	Ap3	33.53461	−80.33138
San Antonio Ditch, TX	Ap6	29.499075	−98.576575
Lake Miccosukee, FL	Ap6	30.52905	−83.98048
Poverty Point, LA	Ap6	32.53	−91.49
Lake Martin, AL	Ap6	32.79806	−85.8197
Anguilla, MS	Ap6	33.0231	−90.8475
Aberdeen Lake, MS	Ap6	33.84	−88.508056
Nickajack, AL	Ap6	34.832464	−87.322239
Germantown Greenway, TN	Ap6	35.1173	−89.8201
333 Cove, AR	Ap6	35.3204	−93.214
Lansbrook Lake, OK	Ap6	35.561083	−97.6231927

### 2.3 Haplotyping

Alligatorweed populations used for this experiment were the same as reported by Williams et al. (2020). To determine haplotype, three apical meristems were collected from each culture, dried in silica gel, then DNA was extracted from all samples (*n* = 78) using the IBI Scientific MINI Genomic DNA kit (Plants) (Dubuque, Iowa) as per the manufacture's instructions. Plants were genotyped using primers developed around mononucleotide repeats in three chloroplast (cpDNA) regions (*rpL16*, *trnS‐G*, *trnF* intron/*trnL‐F* spacer) using previously described methods (Williams et al., 2020). Plants were assigned haplotypes (Ap 1–6) based on the combination of repeat lengths from these three cpDNA regions. For this experiment, we chose replicate populations of the most common haplotypes Ap1, Ap3, and Ap6 (Table 1).

### 2.4 Experimental setup

To test haplotype response to combinations of nitrogen (N) and phosphorus (P), we conducted a three‐way factorial randomized block design experiment with three replicates of each nutrient treatment × population combination. Plants were grown hydroponically in a greenhouse at the US Army Engineer Research and Development Center, Vicksburg, Mississippi, the United States. The day of experimental setup, 15 stems from each alligatorweed population (26 populations total; 11 Ap1, 5 Ap3, 10 Ap6) were clipped just past the third node and floated in reverse‐osmosis (RO) filtered water. The lowest pair of leaves were removed to expose the node and promote rooting, and then the plants were blotted dry before initial weights were recorded. All stems used as starting material had four fully expanded leaves and were approximately 15 cm in length. At planting, plant propagules were placed individually into net pots (12.7 mm diameter) filled with washed expanded clay rocks (8–16 mm diameter). Net pots were placed within white 4‐L polyethylene food containers, and RO water was added to each container. The location of containers was randomized among five greenhouse tables. Randomization was stratified such that each population × nitrogen × phosphorus combination occurred in each of five experimental blocks (tables). Temperature in the greenhouse was maintained between 15 and 25°C, and we used 60% shade cloth on the greenhouse for the duration of the experiment.

Four nutrient solutions were prepared, each containing a combination of low or high nitrogen or phosphorus (Table 2). High nitrogen or phosphorus concentrations were based on standard Hoagland's recipe (200 mg/L N; 40 mg/L P), and low concentrations were 2% (nitrogen) or 1% (phosphorus) of the high concentration. These concentrations were chosen to span those used previously to measure nutrient response of *A. philoxeroides* (Zhang et al., 2017) and other rooted wetland invaders (e.g., Butomus umbellatus; Harms, Cronin, & Gaskin, 2021; Manolaki et al., 2020). All other macro‐ or micronutrients were consistent between solutions. After 1 week of rooting, water was emptied, and 1 L of each respective nutrient solution was added to the containers. The entire volumes of nutrient solutions were replaced weekly for the duration of the experiment, 5 weeks total.

**TABLE 2:** Estimated elemental concentrations (mg/L) in experimental solutions.

Element	Experimental solution			
	High N‐high P	Low N‐high P	Low N‐low P	High N‐low P
N	201	4	4	200
P	40	43	0.4	0.4
K	205	200	197	201
Mg	30	29	29	30
Ca	98	103	98	98
S	126	135	165	130
Cl	109	109	119	108.7
N:P	5	0.1	10	500

The experiment was harvested after 5 weeks, at which time measurements were taken. To measure leaf toughness, two leaves per plant were randomly chosen, placed on a round PVC surface with a 10‐mm hole, then a force gauge was used to measure the force (in pounds) required to push a 9‐mm‐diameter cylindrical rod through the leaf. Length of the main stem, the number of branches on the main stem, and sum total length of branches per stem were measured. Because *A. philoxeroides* produces hollow stems under flooded conditions, and stems are the pupation site of *A. hygrophila* (Maddox et al., 1971), stem diameter and stem wall thickness were measured at the thickest point of each stem using a digital caliper. Two leaves from each plant were collected separately, weighed, and then used in the *A. hygrophila* development assay described in the next section. The remaining leaves were separated from stems, weighed, and placed in paper bags for drying. Dry weights of the leaves removed for the *A. hygrophila* feeding assay were estimated from the dry‐to‐wet weight ratio of the remaining leaves at harvest, then added to aboveground biomass measurements for each plant. Similarly, stems and roots (belowground plant parts) were separated and rinsed with reverse osmosis water, then placed in paper bags for drying. With the exception of leaves used in the development assay, all plant tissues were placed in forced‐air drying ovens and dried at 60°C until constant weight was reached. Once dry, plant parts were weighed to the nearest 0.01 g.

In addition to measurements taken at harvest, some plant traits were calculated. To assess plant growth, total biomass (sum of below and aboveground biomass) and shoot: root ratio (summed stem and leaf biomass divided by root biomass) were calculated. To describe plant architecture, we calculated branching intensity as the ratio of summed branch lengths to main stem length. Leaf dry matter content (DMC) was determined by dividing leaf dry biomass by leaf fresh biomass. Tissue chemistry analyses were performed at the Louisiana State University Agricultural Chemistry Laboratory, Baton Rouge, LA, the United States. Percent leaf nitrogen was determined by the modified Dumas method (CN 628 Dumas Analyzer; LECO) and percent tissue phosphorus was determined by inductively coupled plasma (ICP) mass spectrometry (ARCOS; SPECTRO Analytical Instruments; Jones Jr. & Case, 1990).

### 2.5 Larval development assay

In addition to plant measurements described above, we evaluated the role of nutrients in *A. philoxeroides* herbivore resistance with a short‐term feeding assay. Prior to biomass harvest in the above experiment, two leaves were excised from each plant (*n* = 720 leaves, 24 per population) and used to assess short‐term *A. hygrophila* larval development. Beetles originated from field‐collected individuals at the Blind River, Louisiana, the United States, in November 2019 and were reared through several generations in the laboratory before being used in the experiment. One week prior to the development experiment, male and female *A. hygrophila* were collected, placed as pairs in cups with a single alligatorweed leaf, sealed, and then left at 23°C for several days. Two days before the experiment, egg masses were collected and monitored for egg hatch. Upon emergence, a single *A. hygrophila* neonate was placed in a 30‐mL plastic cup on a leaf collected from alligatorweed plants (described above) grown in different combinations of available nitrogen and phosphorus. Cups were sealed and placed in a random location in trays and incubated at 23°C and 14:10 light: dark for 72 h in a plant growth chamber (E‐41L2; Percival Scientific, Perry, Iowa). The holding temperature was chosen to be within the optimal (23–25°C) range suitable for the development of *A. hygrophila* (Harms & Cronin, 2019; Stewart et al., 1999). After 72 h, larvae were recovered and weighed to the nearest 0.0001 g with an analytical balance (PG403‐S; Mettler Toledo). Because we used excised leaves and because plants were not previously fed on, we functionally investigated the constitutive defense of *A. philoxeroides* plants under varying nutrient regimes.

### 2.6 Statistical approach

To examine whether there were genetic‐based differences in nutrient responses between alligatorweed haplotypes (Ap1, Ap3, Ap6), we used the Akaike information criterion adjusted for small sample size (AICc) to select the most informative mixed model (Proc MIXED; SAS 9.3, SAS Institute, Cary, NC) from the full set of candidate models (Burnham & Anderson, 2003). Candidate models were constructed from the simplified full model (haplotype, nitrogen level, phosphorus level, and all two‐way interactions) with the constraint that an interaction term was included only if their main effects were also included in the model. In all models, *A. philoxeroides* population (nested within haplotype) was included as a random variable, location within the greenhouse was a blocking factor, and initial fresh weight of propagules was included as a covariate. Also, because latitudinal clines in growth, reproduction, or herbivore defense traits may form in large‐scale invasions (Cronin et al., 2015; Liu et al., 2021), we included source latitude of each population as a possible explanatory variable. Response variables for models were plant total dry weight (DW), shoot: root ratio, branching intensity, stem wall thickness, stem diameter, leaf dry matter content (DMC), leaf toughness, percent leaf nitrogen, percent leaf phosphorus, and *A. hygrophila* larval weight. To meet parametric assumptions of the model, larval weight was natural‐log transformed prior to analysis.

The top model had the lowest AIC of all candidate models, and ΔAICc was calculated as the difference between the top model and all others. Models with AICc <2 were considered to have substantial support (Burnham & Anderson, 2003). Akaike weights are also reported, which represent the relative likelihood that the model is the best given the data and other candidate models (Burnham & Anderson, 2003). We used the package MuMIn in R to assess the AICc best model by computing the proportion of variance explained as marginal and conditional *R* ^2^ (Meyerson et al., 2020; Nakagawa & Schielzeth, 2013). Marginal *R* ^2^ is the variance explained by fixed factors and conditional *R* ^2^ is the variance explained by the model (i.e., fixed and random effects combined). To ease interpretation and discussion of differences in treatment means, we estimated least‐squares means (back‐transformed, if necessary) based on the most‐likely model for each response variable and present those graphically. Additionally, because we were specifically interested in plastic responses of haplotypes to nutrients, we calculated effect sizes (Hedge's g) of treatments by haplotype (Borenstein et al., 2011; Ellison et al., 2014).

Plasticity was calculated as Hedge's g for all measured or calculated variables to quantify the direction and magnitude of plant response to nitrogen or phosphorus treatments. We first calculated population‐level least‐squares mean response to nitrogen or phosphorus independent of the other nutrient. We then calculated Hedge's *g* (*J*‐corrected Cohen's *d*) (Borenstein et al., 2011; Davidson et al., 2011) from population means for each response variable and nutrient treatment combination. We quantified treatment effects in this way because it allows a comparison between multiple traits that have been standardized in units of standard deviation (Cook‐Patton & Agrawal, 2011). First, *d* was calculated as:

$$d=\frac{{\text{Mean}}_{\max}-{\text{Mean}}_{\min}}{{\mathrm{SD}}_{\text{pooled}}}$$

where Mean_max_ and Mean_min_ were the maximum and minimum mean response values for each nutrient and population. Pooled standard deviation was calculated as in Borenstein et al. (2011):

$${\mathrm{SD}}_{\text{pooled}}=\frac{\sqrt{\left(n_{1}-1\right){{\mathrm{SD}}_{\max}}^{2}+\left(n_{2}-1\right){{\mathrm{SD}}_{\min}}^{2}}}{n_{1}+n_{2}-2}$$

We then applied the *J* correction for small sample size using a sample size of six per treatment combination (Borenstein et al., 2011):

$$J=1-\frac{3}{\left(4\mathrm{df}-1\right)};$$

g=J*d.

Once we calculated population‐level *g* (plasticity), we further compared plasticity of *A. philoxeroides* haplotypes to nitrogen or phosphorus treatments. To do this, we used mixed models with *g* as the dependent variable, haplotype as the fixed effect, and population as a random effect. All statistical analyses were performed in SAS version 9.4 (SAS Institute).

## 3 RESULTS

### 3.1 Response of *A. philoxeroides* haplotypes to elevated nutrients

**FIGURE 1:**
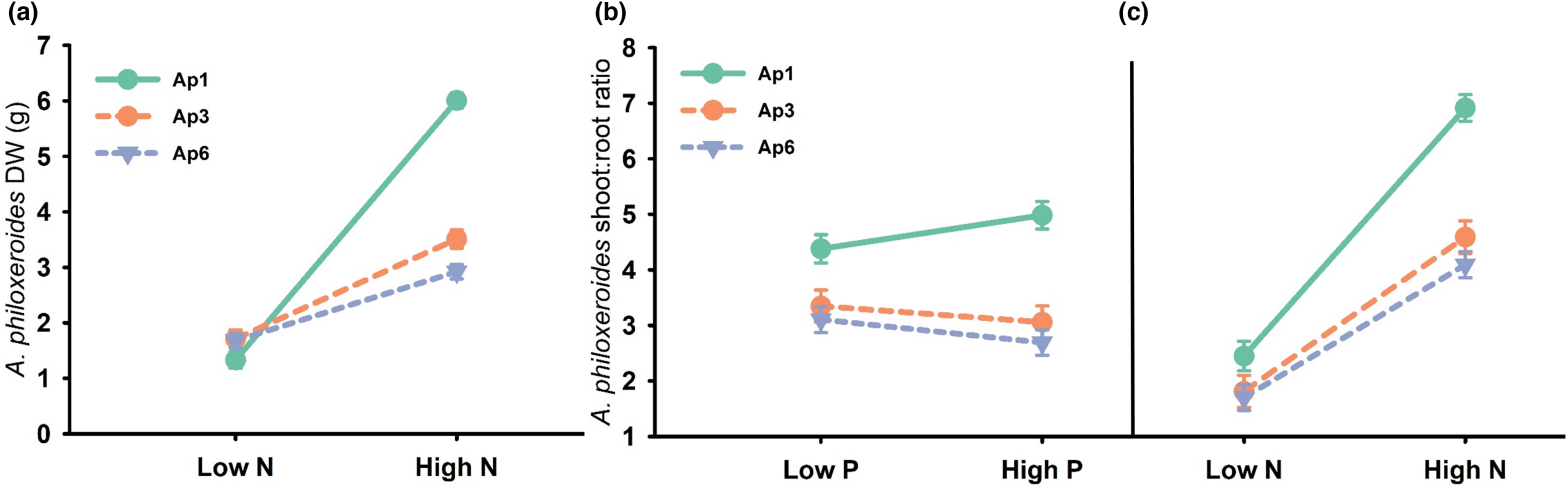
Influence of nutrients on biomass production (g DW; least squares mean ± SE) (a) and allocation (b, c) of *A. philoxeroides* haplotypes. These interactions were identified as influential based on model selection. Error bars obscured due to small size.

In nearly all measured response variables, plant haplotype was a significant explanatory variable based on AICc model selection (Table 3). *Alternanthera philoxeroides* haplotype, both nutrients, latitude, and their interactions were influential in explaining biomass production and allocation. Although four candidate models received support (ΔAICc ≤2) for dry weight (DW) biomass, the top model included only haplotype, nitrogen, phosphorus, and the haplotype × nitrogen interaction (*R* ^2^_(m)_ = 0.80; *R* ^2^_(c)_ = 0.82). Total biomass for all three haplotypes was similar at 2 mg/L N, but 71% and 105% greater for Ap1 at 200 mg/L than Ap3 and Ap6 haplotypes, respectively (Figure 1a). Shoot: root ratio of *A. philoxeroides* was plausibly explained by two models (Cumulative AICc weight = 0.66) but the top model included haplotype, nitrogen, phosphorus, and the interactions between haplotype and either nutrient. Biomass allocation to aboveground plant parts increased only for the Ap1 haplotype with phosphorus (Figure 1b; Ap1: 14% increase, Ap3: 9% decrease, Ap6: 13% decrease) but increased with N for all haplotypes (Figure 1c; Ap1: 182%, Ap3: 153%, Ap6: 141% increased).

**TABLE 3:** Top best‐fit models for each dependent variable, based on AICc selection procedure.

Variable	Model	df	AICc	ΔAICc	Likelihood	AICc Wt	*R* ^2^_(m)_	*R* ^2^_(c)_
Alligatorweed DW	H + N + P + H * N	4	839.9	0	1	0.18	0.80	0.82
	H + N + P + H * N + N * P	5	840.6	0.7	0.7	0.13		
	H + N + P + H * N + H * P	5	841.2	1.3	0.52	0.09		
	H + N + P + LAT + H * N + H * LAT	6	841.4	1.5	0.47	0.08		
Shoot: root ratio	H + N + P + H * N + H * P + N * P	6	1197.1	0	1	0.33	0.54	0.54
	H + N + P + H * N + H * P	5	1197.1	0	1	0.33		
Branching intensity	H + N + P + H * N + N * P	5	−28.3	0	1	0.63	0.73	0.76
	H + N + P + H * N	4	−26.7	1.6	0.45	0.28		
Stem wall thickness	H + N + H * N	3	−395.9	0	1	0.9	0.60	0.68
Stem diameter	H + N + H * N	3	−462.7	0	1	0.79	0.72	0.90
Dry matter content	Intercept	1	−1386.8	0	1	1	0.01	0.05
Leaf toughness	H + N + H * N	3	−555.1	0	1	0.88	0.37	0.39
Leaf percent N	H + N + P + H * N + N * P	5	430	0	1	0.35	0.89	0.91
	H + N + H * N	3	431.1	1.1	0.58	0.2		
	H + N + P + H * N + H * P + N * P	6	431.6	1.6	0.45	0.16		
Leaf percent P	N + P + N * P	3	179.3	0	1	0.94	0.30	0.33
Ln(*Agasicles* weight)	N + P + N * P	3	562.6	0	1	0.35	0.10	0.12

*Note*: Model goodness‐of‐fit (*R* ^2^_(m)_, *R* ^2^_(c)_) for each variable is shown for the top model only.

Abbreviations: AICc Wt., AICc weight; AICc, Akaike Information criterion adjusted for small sample size; df, degrees of freedom; DW, dry weight; H, Haplotype; N, Nitrogen; P, Phosphorus; *R* ^2^_(c)_, conditional *R* ^2^; *R* ^2^_(m)_, marginal *R* ^2^; ΔAICc, difference between AICc of the model and AICc of the top model.

**FIGURE 2:**
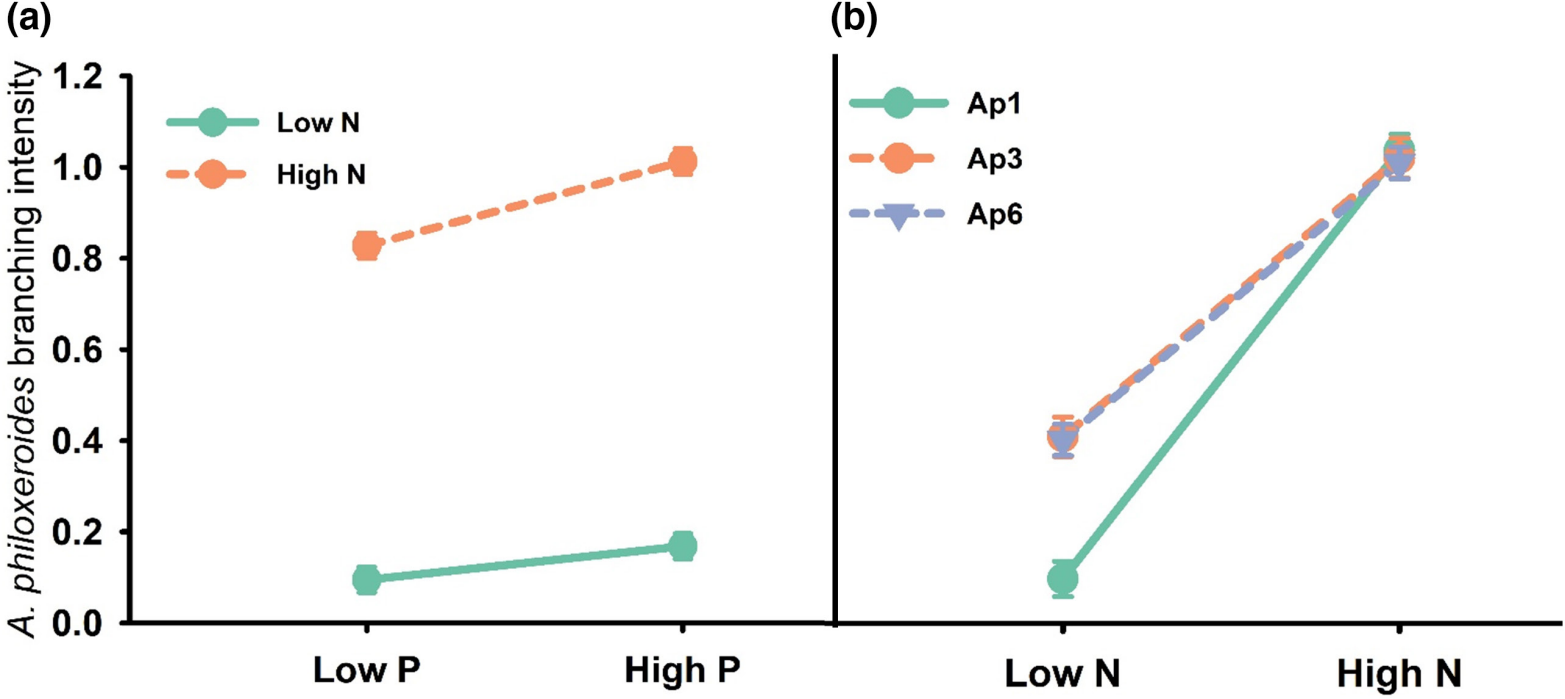
Influence of the nitrogen × phosphorus interaction (a) and the haplotype × nitrogen interaction (b) on branching intensity (least squares mean ± SE). These interactions were identified as influential based on model selection. Error bars obscured due to small size.

Branching intensity was best explained by two models (cumulative AICc weight = 0.91) but the top model included haplotype, nitrogen, phosphorus, haplotype х nitrogen interaction, and the nitrogen х phosphorus interaction (Table 2; AICc = −28.3; AICc weight = 0.63). Branching intensity was influenced by the interaction between N and P and increased 22% from low to high P at low levels of N and 78% for high N treatments (Figure 2a). Branching intensity was also higher for Ap3 and Ap6 haplotypes at low N only (Figure 2b).

**FIGURE 3:**
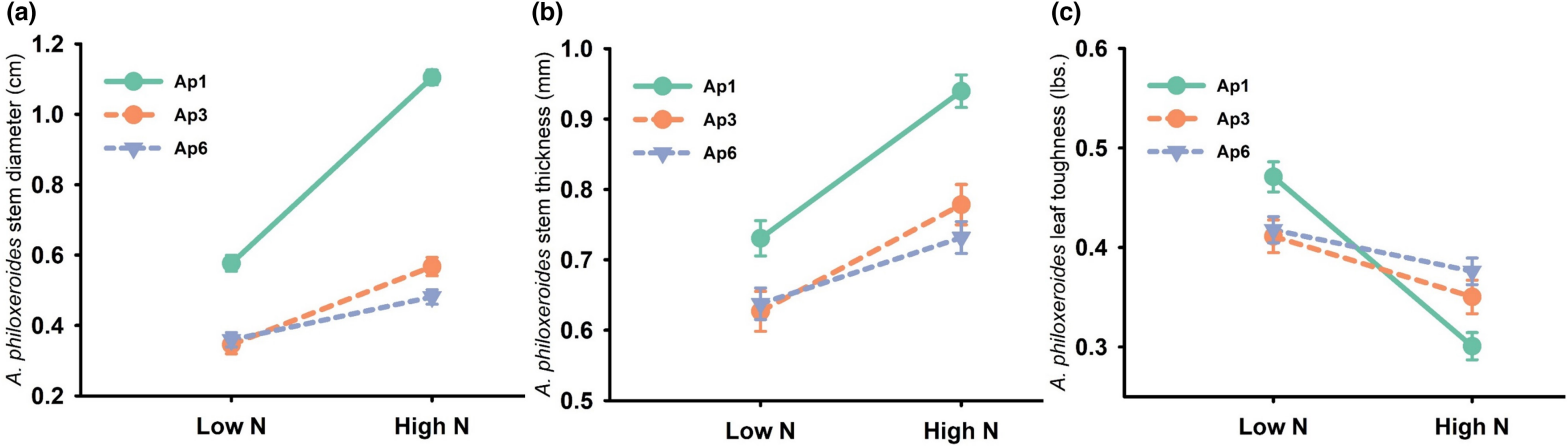
Influence of the haplotype х nitrogen interaction on *A. philoxeroides* stem diameter (a), stem wall thickness (b), and leaf toughness (least squares mean ± SE) (c). These interactions were identified as influential based on model selection. Error bars obscured due to small size.

Variation in stem diameter, stem wall thickness, and leaf toughness variables were all explained by haplotype, nitrogen, and the haplotype х nitrogen interaction (Table 1). Stem diameter was consistently higher for haplotype Ap1 and increased 92% from low to high levels of N, whereas Ap3 and Ap6 stem diameter increased 64% and 33% over the same N treatments (Figure 3a). Stem wall thickness followed a similar pattern—stem walls of haplotype Ap1 plants were consistently thicker than other haplotypes and increased with N 29% (Ap1), 24% (Ap3), and 14% (Ap6) (Figure 3b). *Alternanthera philoxeroides* leaf toughness decreased with increasing N, although the decrease was greatest for Ap1 plants (Figure 3c). Dry matter content was not influenced by treatment variables in the experiment and was best explained by the intercept‐only model (AICc = −1386.8; AICc weight = 1.0).

**FIGURE 4:**
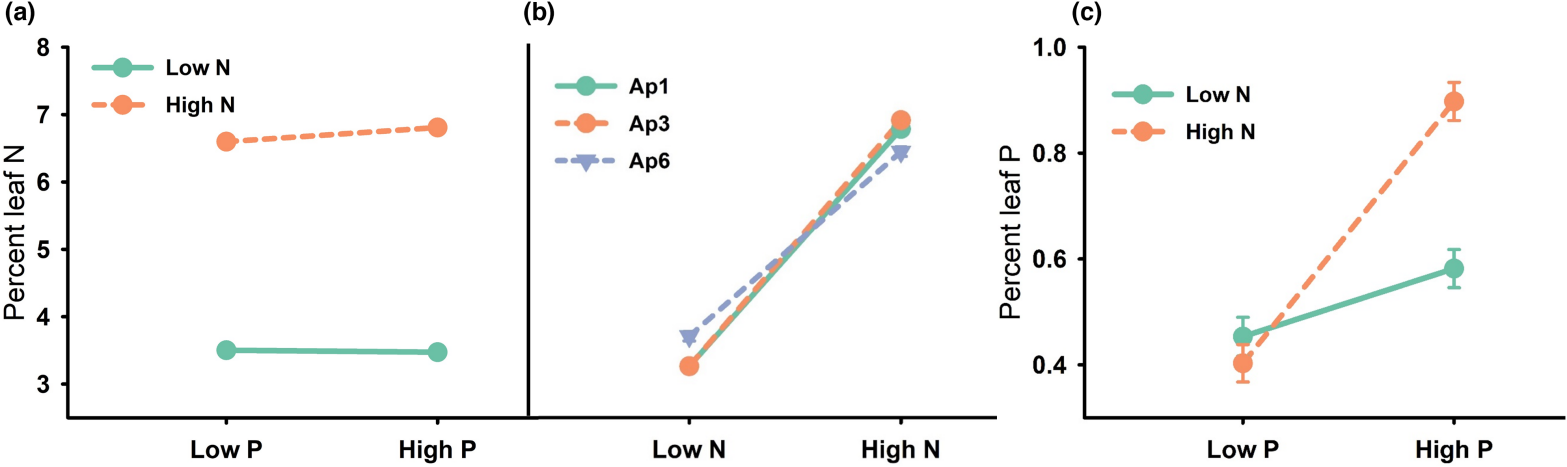
Influence of nitrogen × phosphorus interaction (a), haplotype × nitrogen interaction (b) on *A. philoxeroides* leaf nitrogen and nitrogen × phosphorus interaction on leaf phosphorus (c) (least squares mean ± SE). These interactions were identified as influential based on model selection. Error bars obscured due to small size.

Variation in percent leaf N was explained by three plausible models (cumulative AICc weight = 0.71) but the top model included haplotype, nitrogen, phosphorus, the haplotype × nitrogen interaction, and the nitrogen × phosphorus interaction (AICc = 430.0; AICc weight = 0.35). Percent leaf nitrogen increased slightly from low to high levels of phosphorus but only in the high nitrogen treatment (Figure 4a). Leaf nitrogen increased from low to high nitrogen treatments for all haplotypes, but the increase was greatest for Ap1 and Ap3 (Figure 4b). Variation in percent leaf phosphorus was best explained by a single model; percent leaf phosphorus was influenced by nitrogen, phosphorus, and their interaction (Figure 4b; AICc = 179.3; AICc weight = 0.94). Percent leaf phosphorus increased with phosphorus treatments, but the magnitude of increase was highest in high nitrogen treatments, regardless of haplotype (Figure 4c; 123% increase from low to high phosphorus).

**FIGURE 5:**
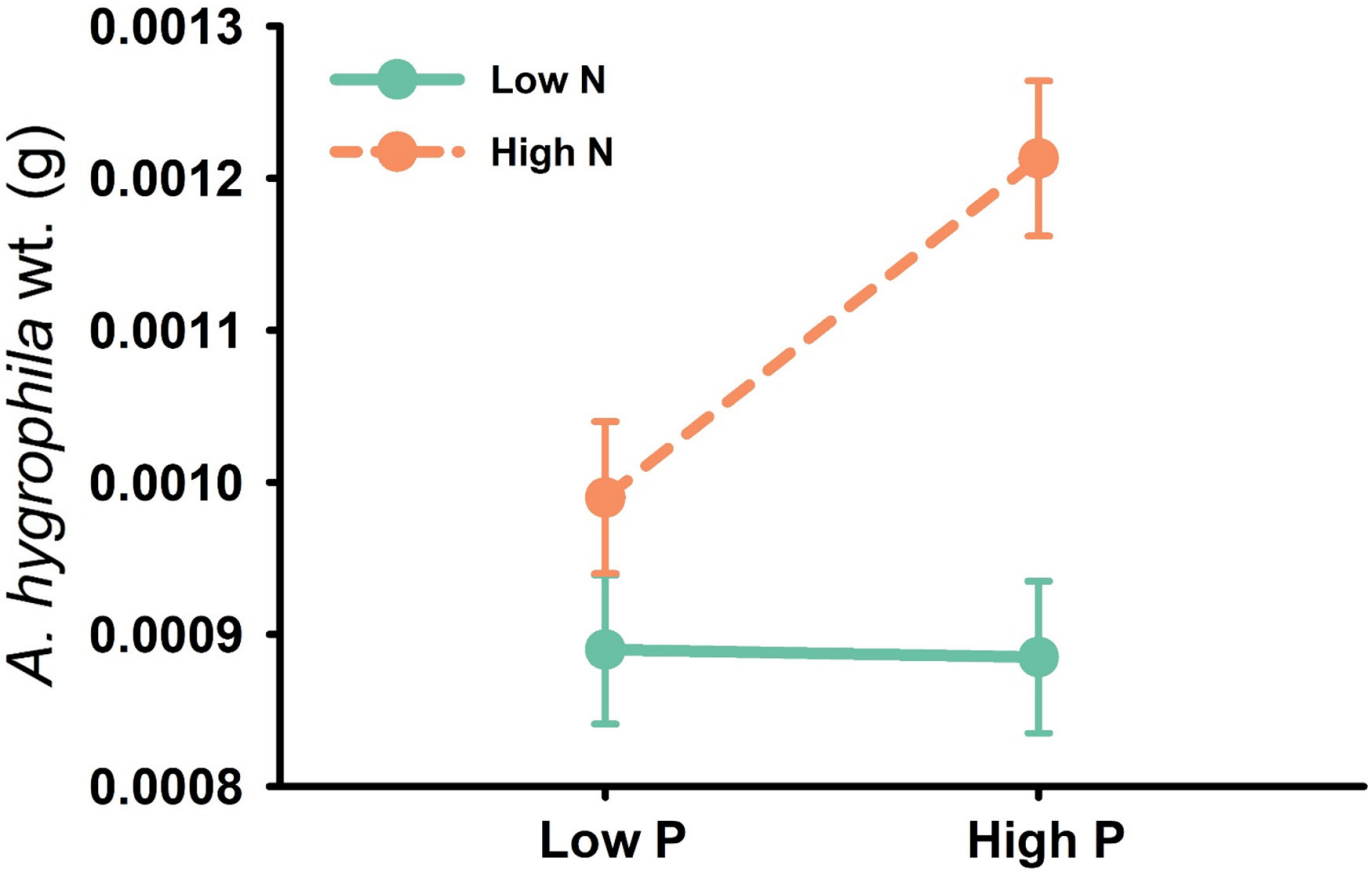
Influence of the nitrogen × phosphorus interaction on resistance to herbivory (*A. hygrophila* larval weight; least squares mean ± SE of untransformed data). The interaction was identified as influential based on model selection.

In the short‐term feeding assay, *Agasicles hygrophila* weight was influenced by the nitrogen × phosphorus interaction (Table 1; AICc = 562.6; AICc weight = 0.35). Regardless of *A. philoxeroides* haplotype, larval weight was greater overall in the high nitrogen treatment, increasing 23% from low to high phosphorus (Figure 5).

### 3.2 Haplotype plasticity to nutrient treatments

**FIGURE 6:**
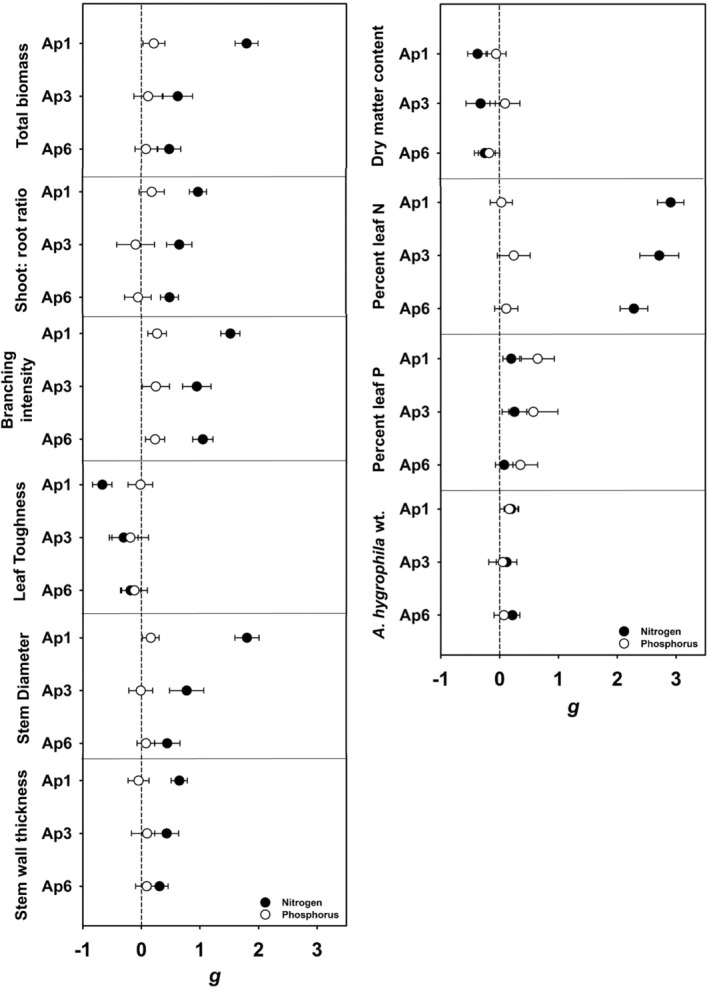
Plasticity (*g*; mean ± 95% CI) of *A. philoxeroides* haplotypes in response to nitrogen (black circle) or phosphorus (white circle) treatments.

We detected differences in plasticity to nutrients between *A. philoxeroides* haplotypes in seven of 10 N‐related responses (total biomass, shoot: root ratio, branching intensity, stem thickness, stem diameter, leaf toughness, and percent foliar nitrogen), as indicated by significant differences in effect size between haplotypes (nonoverlapping 95% confidence intervals in Figure 6). Ap1 plants were more plastic in response to nitrogen for nearly all measured variables. Plasticity to nitrogen for total biomass was 190% higher for Ap1 plants than Ap3, and 282% higher than Ap6. Similarly, plasticity in shoot: root ratio (Ap1 *g* = 0.97 ± 0.07; Ap3 *g* = 0.65 ± 0.10; Ap6 *g* = 0.49 ± 0.07) and branching intensity (Ap1 *g* = 1.52 ± 0.08; Ap3 *g* = 0.95 ± 0.12; Ap6 *g* = 1.05 ± 0.08) were significantly greater in Ap1 plants. Plasticity in stem thickness (g = 0.65 ± 0.07) and stem diameter (g = 1.81 ± 0.10) for Ap1 plants was higher than other haplotypes. Ap1 plants displayed two times the plasticity for leaf toughness than Ap3 plants and three times that of Ap6 plants (Ap1 *g* = −0.67 ± 0.08, Ap3 *g* = −0.303 ± 0.12, Ap6 g = −0.19 ± 0.08) but only 7% more plasticity than Ap3 and 27% more than Ap6 in percent leaf nitrogen in response to nitrogen treatments. Response of percent leaf phosphorus, DMC, and resistance to herbivory (*A. hygrophila* weight) were similar between haplotypes in response to nitrogen. In contrast to the strong response to nitrogen, we detected no differences between haplotypes in plasticity of any variables to phosphorus treatments (Figure 6).

## 4 DISCUSSION

For invaders with multiple introduced genotypes, determining how environmental variation contributes to their success, and whether the response to the environment varies by genotype, can improve predictions about which invaders (or genotypes) will become invasive (i.e., using a trait‐based approach; Bhattarai et al., 2017; Davidson et al., 2011; Weinig et al., 2007) and guide decision‐making around management actions (Gaskin et al., 2011; Thum, 2018). There are a growing number of invaders for which it is clear that management should be tailored at the subspecific level (Blossey & Casagrande, 2016; Croy et al., 2020; Harms, Shearer, et al., 2020; Harms, Williams, & Purcell, 2021), and this work further supports continuation of that research direction. How genotypes of introduced species differ in their tolerance or response to environmental variation is key to distinguishing invasive traits, and thus modeling their distribution and impacts in the invaded range.

Our results demonstrate that there is significant variation in *A. philoxeroides* haplotype‐specific responses to elevated nutrients. We did not directly test whether increased plasticity is adaptive for *A. philoxeroides*; however, for a plant that reproduces primarily by clonal fragmentation, we provided evidence for increased fitness through several proxies, including variation in biomass production and branching intensity related to haplotype and nutrient level. Increased branching, for example, may lead to increased matting on the water surface and more propagules available for dispersal upon disturbance. This is one of only a few studies to examine intraspecific variation in plant traits of *A. philoxeroides* in the United States, and joins a growing number of studies in the invasion biology literature that demonstrate (1) intraspecific variation in response to biotic or abiotic environmental variables within an invading species (Harms, Cronin, & Gaskin, 2021; Lavergne & Molofsky, 2007), and (2) the potential importance of phenotypic plasticity for invader success (Bhattarai et al., 2017; Castillo et al., 2014; Chevin & Lande, 2011; Eller & Brix, 2012; Geng et al., 2016).

### 4.1 Nutrients and management

In the United States, *A. philoxeroides* has a large geographic distribution that includes the entire southeastern region and California. Of the six recognized haplotypes in the United States, Ap1 has the broadest latitudinal distribution, collected from southern Florida and Texas north to Arkansas and North Carolina (Williams et al., 2020). *Alternanthera philoxeroides* haplotype Ap1 had a similar biomass response at low nutrients as Ap3 and Ap6 but the greatest response to elevated nutrients by most measures, which suggests that it is well‐adapted to a range of nutrient conditions. The increased response of invasive weeds over native species to high resource availability can exacerbate the displacement of native species (e.g., Leishman & Thomson, 2005; Richardson & Pyšek, 2006; Rickey & Anderson, 2004), which may explain the broad geographic distribution of Ap1. Also, although there is substantial overlap in the areas where *A. philoxeroides* haplotypes occur, there is often only a single haplotype within a site (Williams et al., 2020). Whether there is some spatial segregation based on nutrient conditions is unknown, but the differences in haplotype response to nutrients may explain some of that variation. Ap1 had the highest biomass response to elevated nutrients, largely in the form of shoot production and branching, suggesting that a competitive advantage over other species may stem from shading in addition to nutrient use. In contrast, Ap6 plants grew slower but allocated a larger proportion of biomass to roots overall and produced more biomass and higher branching in low nutrient treatments, which may be advantageous and explain marginally greater assimilation of N under low nutrient conditions (Figure 4b). In addition, the current study used only the three most common *A. philoxeroides* chloroplast haplotypes to evaluate variation in plasticity, but there are a number of other rare ones in the invaded range. For example, California populations so far consist of only Ap2 and Ap4 haplotypes, which are only known to occur in one other state each (Ap2 in Georgia, Ap4 in Arkansas) (Williams et al., 2020). Ap5 was only detected in a single location in Louisiana and may not occur anywhere else in the United States. Investigation into other, rare haplotypes may provide important insights into their ecology and potential to become more widespread and invasive.

We did not find differences in herbivore resistance between *A. philoxeroides* haplotypes, but the influence of plant nutrition was clear. Differences in *A. hygrophila* development time on narrow and broad‐leafed *A. philoxeroides* have been demonstrated (Pan et al., 2012, 2013) and had we allowed development to continue in our experiment, we may have observed similar differences between haplotypes Ap1 and Ap6, which are the morphological analogs to narrow and broad‐leafed *A. philoxeroides* (Williams et al., 2020). However, prolonging the assay would have increased the volume of biomass removed from the treatments, delaying collection, and potentially altering the final plant measurements. Thus, the window for development was intentionally kept short.

Despite the short development time, we found the influence of elevated nutrients on several plant traits that influence successful biological control. For example, at elevated nitrogen treatments, foliar nitrogen increased, DMC decreased, and leaf toughness decreased—all results which should improve the development of the foliar‐feeding *A. hygrophila*. In fact, previous work demonstrated that, at high nitrogen levels, larval development time decreased and larval survival increased (Harms & Cronin, 2019). Although larval development would be expected to accelerate on all haplotypes, Ap1 is most likely to have the largest influence on development time, if development was allowed to continue through eclosion. Stem diameter was largest overall for Ap1 plants, and also responded most to increased nitrogen. However, stem wall thickness also increased in response to nitrogen, which may have a confounding impact on larval *A. hygrophila*, if given sufficient time to develop and pupate. The diameter of *A. philoxeroides* stems is important for *A. hygrophila* pupation, a feature which has been suggested to possibly limit persistence of agents in some populations, particularly those growing in terrestrial habitats (Ma & Wang, 2004; Pan et al., 2011).

Herbicide control of *A. philoxeroides* can be highly effective, especially in areas where biological control does not provide adequate control or where agents have not been introduced (Clements et al., 2017). Although herbicide tools are available, which to use and where to use them largely depends on adjacent land‐use and whether the plants are growing in flooded or dry habitat (Dugdale & Champion, 2012). To our knowledge, the roles of plant genotype and nutrient availability on herbicide efficacy have not been addressed for any emergent or floating weeds in the United States, though evidence for variation in herbicide susceptibility among genotypes of some invaders is mounting (e.g., Chorak & Thum, 2020). Two variables that may influence herbicide effectiveness in *A. philoxeroides* are branching architecture and biomass allocation. The greater the proportion of biomass allocated to roots, the more difficult it is to achieve adequate multiyear control of *A. philoxeroides* (Clements et al., 2017; Schooler et al., 2008). Ap6 plants had the lowest shoot: root ratio overall, regardless of nutrient treatment, and the ratio increased the least from low to high nitrogen or phosphorus. This finding, and that the highest biomass overall and greatest biomass response to increased nutrients occurred for Ap1, suggests that Ap6 plants may be least affected by herbicide application, all other variables held constant. Additionally, increased proportional allocation to aboveground growth under high nutrient conditions may make Ap1 more susceptible to biocontrol and herbicides targeting emergent growth.

It has been suggested that highly plastic species are the most successful invaders because they can tolerate a wide variety of biotic and abiotic conditions (Davidson et al., 2011; Higgins & Richardson, 2014; Richards et al., 2005). Outstanding questions remain about whether species or genotypes have the observed level of plasticity upon introduction, or whether they evolve increased plasticity in novel habitats. The widespread success of *A. philoxeroides* is thought to be largely a result of plasticity to environmental variation (e.g., to water availability; Geng et al., 2016), a pattern that has not differed between native and introduced ranges in earlier studies, and suggests that *A. philoxeroides* did not evolve greater plasticity during invasion in the United States. However, previous studies did not test a genetically diverse subset of populations from the invaded‐range, or make explicit comparisons between introduced genotypes to evaluate the range of plasticity within the invasion. In the current study, we demonstrated high plasticity to nitrogen, but the degree of plasticity varied among measured traits and haplotypes.

Differences in response to elevated levels of nitrogen and phosphorus were observed among common *A. philoxeroides* haplotypes found within their invasive range. It is unsurprising that the haplotype with the greatest degree of plasticity (Ap1) in traits affecting spread and management is also the most prevalent. Although there is limited evidence for differences in efficacy of herbicide and biological control among *A. philoxeroides* haplotypes, these differences have yet to be explicitly explored. There has also not been any investigation into how this plasticity affect intraspecific competition among haplotypes or influences their geographic distributions. More work is needed to fully evaluate conditions that influence *A. philoxeroides* invasion success, and field verification of haplotype distribution in relation to temperature or nutrient responses would improve predictions about locations at risk for future establishment and spread.

## AUTHOR CONTRIBUTIONS

**Nathan E. Harms:** Conceptualization (lead); data curation (equal); formal analysis (lead); funding acquisition (lead); investigation (equal); methodology (equal); project administration (lead); supervision (equal); writing – original draft (lead); writing – review and editing (equal). **Ian A. Knight:** Formal analysis (supporting); investigation (equal); methodology (supporting); writing – review and editing (equal). **A. Blake DeRossette:** Data curation (equal); investigation (equal); writing – review and editing (equal). **Dean A. Williams:** Formal analysis (equal); investigation (equal); methodology (equal); writing – review and editing (equal).

## FUNDING INFORMATION

No external funding was provided for this work.

## CONFLICT OF INTEREST STATEMENT

The authors declare that they have no known competing financial interests or personal relationships that could have appeared to influence the work reported in this paper.

## Data Availability

Dataset is available through Dryad at https://doi.org/10.5061/dryad.v9s4mw71c.
